# Dual‐Energy CT in Breast Cancer: Current Applications and Future Outlooks

**DOI:** 10.1002/pro6.1213

**Published:** 2023-12-01

**Authors:** Shaolan Guo, Tianye Liu, Guobin Qu, Jian Xu, Qingzeng Liu, Qian Zhao, Zhao Bi, Wanhu Li, Jian Zhu

**Affiliations:** ^1^ Shandong First Medical University and Shandong Academy of Medical Sciences Jinan P. R. China; ^2^ Department of Radiation Oncology Physics and Technology Shandong Cancer Hospital and Institute Shandong First Medical University and Shandong Academy of Medical Sciences Jinan P. R. China; ^3^ Shandong Cancer Hospital and Institute Shandong First Medical University and Shandong Academy of Medical Sciences Jinan P. R. China

**Keywords:** Dual‐energy CT, Breast cancer, Quantitative parameters, Clinical diagnosis, Prognosis prediction

## Abstract

Breast cancer is the most prevalent cancerous tumor in women, characterized by different subtypes and varying responses to treatment. The continued evolution of breast cancer diagnosis and management has resulted in a transition from a one‐size‐fits‐all approach to a new era of personalized treatment plans. Therefore, it is essential to accurately identify the biological characteristics of breast tissue in order to minimize unnecessary biopsies of benign lesions and improve the overall clinical process, leading to reduced expenses and complications associated with invasive biopsy procedures. Challenges for future research include finding ways to predict the response of breast cancer patients to adjuvant systemic treatment.

Dual‐energy CT (DECT) is a new imaging technology integrating functional imaging and molecular imaging. Over the past decade, DECT has gained relevancy, especially in oncological radiology. This article proposed a literature review of the application and research status of DECT in breast cancer treatment strategy determination and prognosis prediction.

## Graphical abstract

Dual‐energy CT (DECT) is a new imaging technology integrating functional imaging and molecular imaging. DECT can provide 12 categories of energy parameter image analysis, from which we can obtain multiple parameters to help us find diseases, differential diagnosis and predict prognosis and so on. DECT can be used as a supplementary noninvasive method for breast cancer, and it has great value in the diagnosis, treatment strategy and prognosis prediction of breast cancer in the future.

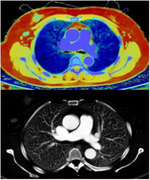

## 1 INTRODUCTION

Breast cancer is a worldwide issue because of its high occurrence rate globally. The incidence of breast cancer has been on the rise in the past 40 years. In recent years (2010‐2019), the incidence rate has increased by 0.5% per year, mainly in early breast cancer and hormone receptor‐positive breast cancer. On the contrary, the decline in breast cancer mortality has slowed down in recent years. ^1^ Over the past few years, advancements in female breast imaging have had a significant impact on the diagnosis, treatment, and prognosis of breast cancer. After the diagnosis of breast cancer, the most direct challenge in patients' treatment strategy is to accurately predict the early efficacy and recommend efficacy‐oriented decision‐making for follow‐up treatment.

DECT is more and more widely used and the usefulness of this technique has been demonstrated in the imaging of tumors in various organs.^2^, ^3^, ^4^ Compared with conventional CT, DECT can display disease information more subtly which is conducive to early detection, early diagnosis, and early effective treatment of diseases. Its application in the field of breast diseases has begun to receive widespread attention.

## 2 DECT: TECHNOLOGY AND PHYSICAL BASIS

**FIGURE 1:**
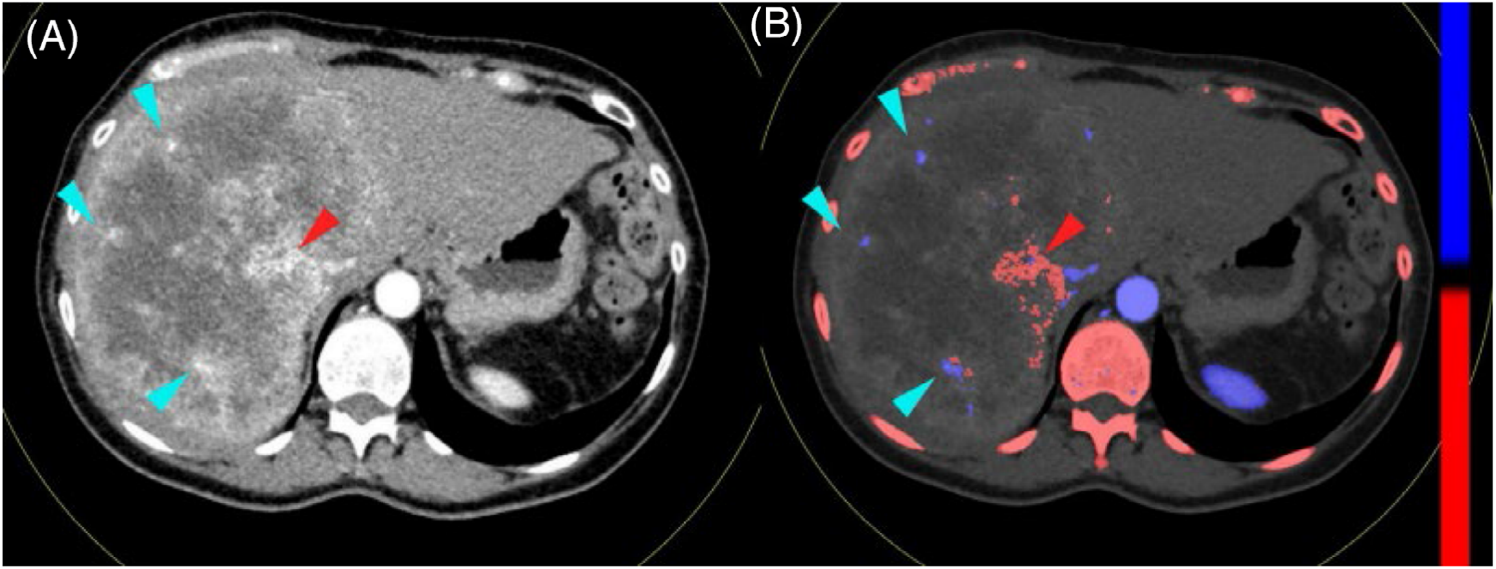
CT and dual‐energy CT. Female, metastasis from colorectal cancer. **A** Mixed image. The enhancing arteries within the lesion (light blue arrowheads) have density similar to intralesional calcifications (red arrowhead). **B** Material labeling. The material decomposition evaluates the different attenuation curves of the basis materials, allowing for material labeling (iodine: blue map and arrowheads; calcium: red map and arrowheads).

**FIGURE 2:**
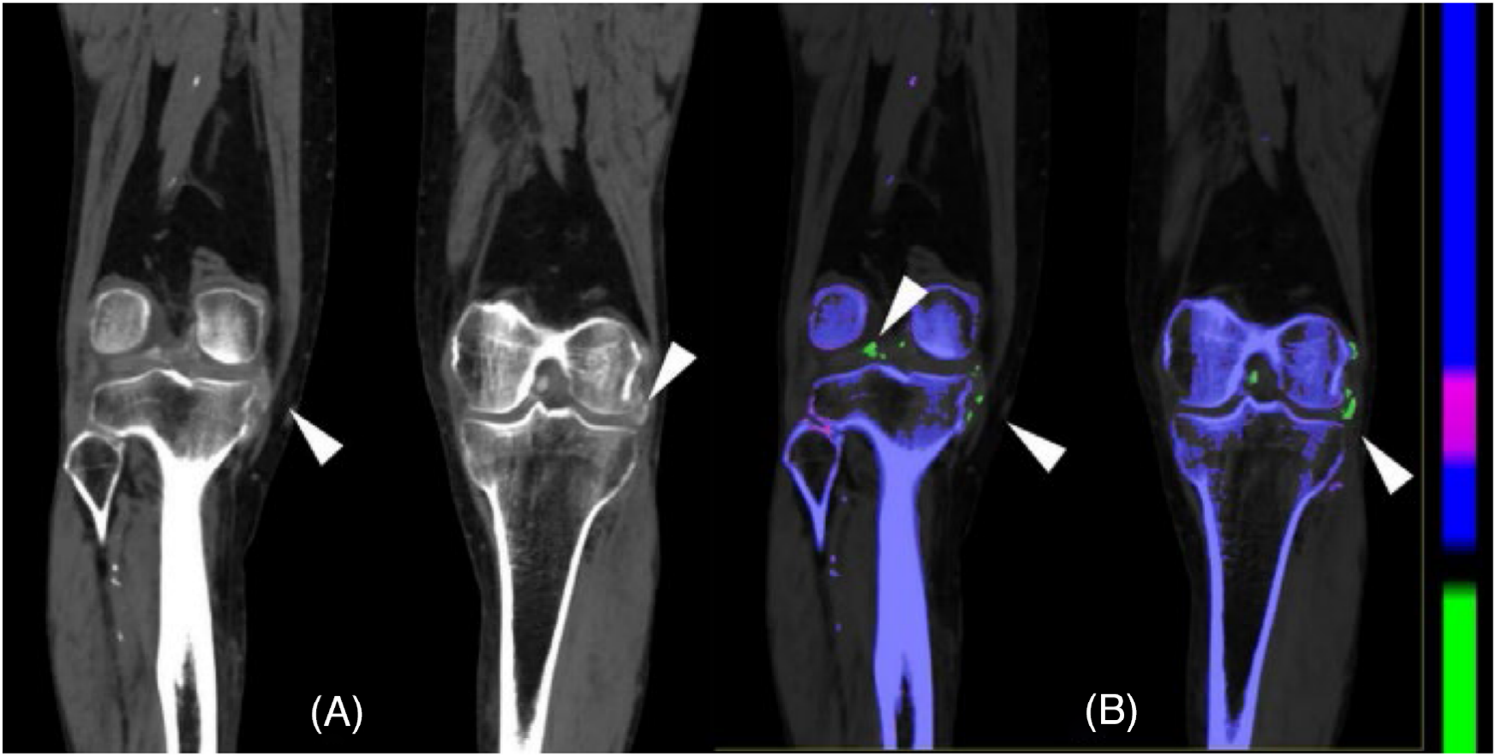
Dual‐source CT. Male, gout. **A** Dual‐energy acquisition, 80/150 Sn kVp, mixed 0.5, coronal; arrowheads on hyperdense deposits on the right and left knees. Material decomposition allows for labeling of monosodium urate crystals in green on coronal (**B**) (arrowheads) with volumetric estimation.

Paraphrased DECT involves capturing CT images using two different energy spectra, allowing for the acquisition of tissue composition data that is not possible with traditional CT scans. The absorption of a photon beam is influenced by the energy of the incoming photons as well as the atomic number and density of the material being penetrated. This is because of the “attenuation phenomenon”: the energy spectrum of X‐ray photons contains information about the composition of their elements after passing through the absorber, resulting in a transition from attenuation‐based imaging to material‐specific or spectral imaging. DECT imaging relies on the ability to distinguish between different energy levels of the beam, allowing for the separation of materials with similar CT attenuation but different atomic numbers. This technique is particularly useful for detecting and measuring the presence of iodine, calcium, or uric acid (Figures 1 and 2).^5^ Various methods can be employed for post‐processing to create iodine maps, virtual non‐contrast images, mixed or weighted images, virtual monochromatic images, and images of specific materials.

## 3 IMAGING EQUIPMENT FOR DUAL‐ENERGY CT IMAGING

**FIGURE 3:**
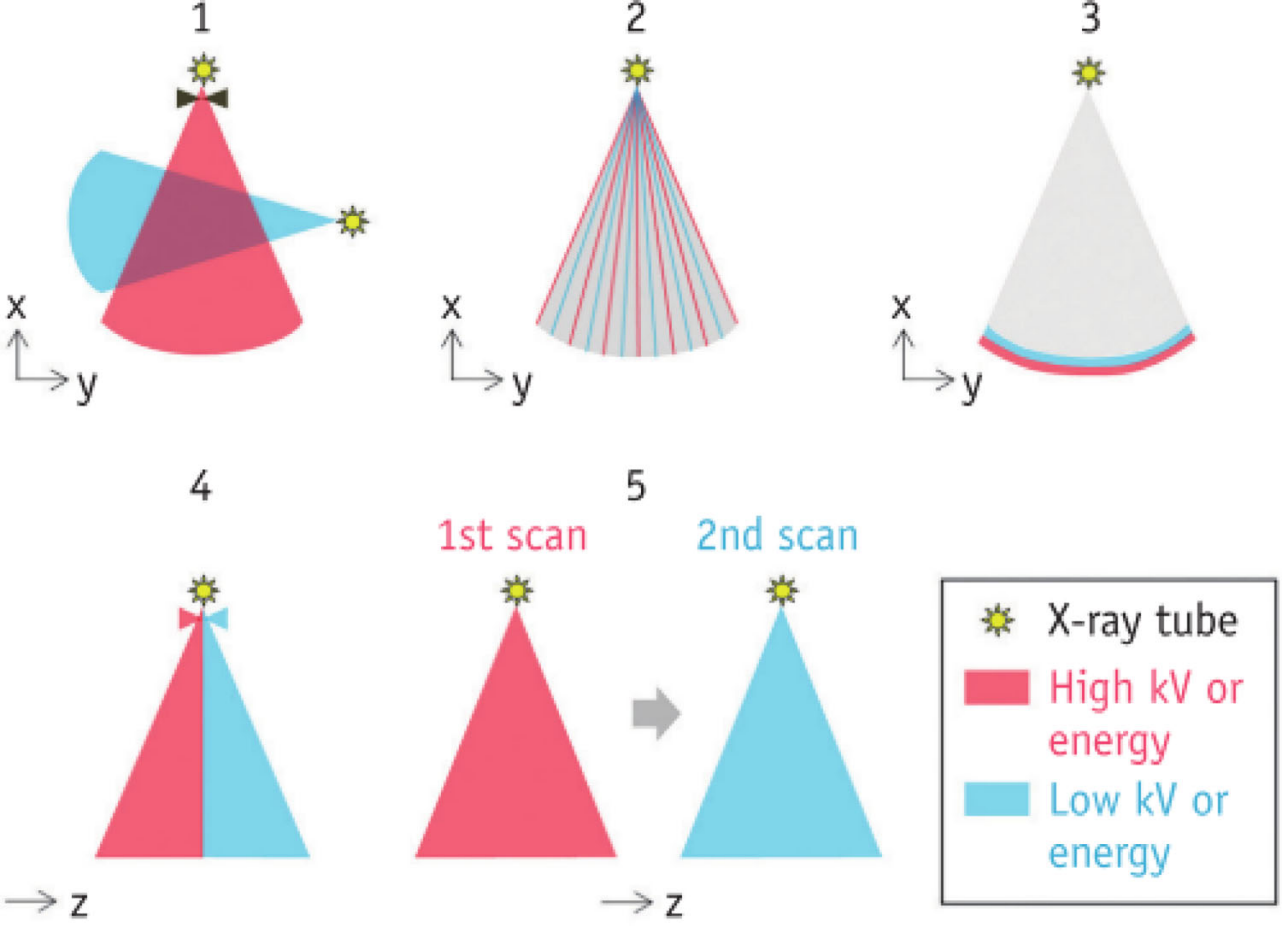
Illustration of five different methods of dual‐energy CT data acquisition. 1 dual tubes with or without beam filtration,2 the fast kV switching of single tubes, 3 dual‐layer detector with single tube, 4 single tube with split filter, 5 single tube with sequential dual scans.

We list five currently available dual‐energy scanning techniques to facilitate understanding and avoid conceptual confusion (Figure 3). ^6^

The first is dual‐source technology, the scanner rotates around the patient and operates different kilovolt settings independently by using two orthogonal X‐ray tubes and corresponding detectors installed in the scanner.^7^ Another technique uses the fast kilovolt (kV) switching of single tubes (usually smaller than 0.5ms). This technique involves quickly changing the tube voltage between 80 and 140kVp, and gathering two sets of projection data for later use in the dual energy reconstruction algorithm based on projections.^6^ The third technique involves using a dual‐layer detector to capture both low‐energy and high‐energy photons from the same imaging beam, thereby enabling the detection of spectral information. The fourth is a single tube with a split filter, which so‐called double‐beam technology. By employing a specialized filter, the X‐ray beam can be effectively divided into two distinct energy spectra at the kilovolt source. Subsequently, various geometric sections of the detector can be exposed to these separate energy spectra.^8^ The fifth method involves using a single tube with sequential Dual Scans. In this approach, dual‐energy CT data is obtained by performing spiral or sequential scans twice in succession, using two different tube voltages, typically 80 and 140 kVp.^6^ DECT imaging employs a technique where the dose is split into two energies during the acquisition process, resulting in a dose level that is roughly equivalent to that of a single energy scan.^9^ Consequently, the acquisition of DECT images does not necessarily lead to an increase in the radiation dose administered to the patient.

## 4 MATERIAL QUANTITATIVE IMAGING AND MONO‐ENERGETIC DECOMPOSITION

DECT can provide 12 categories of energy parameter analysis, this article only introduces a few commonly used parameters of breast diseases.

### 4.1 Conventional CT image

DECT can get conventional CT images every time it scans. For example, the principle of double‐layer detector is that each layer detector absorbs high‐and low‐energy photons in the same beam respectively to generate two sets of high‐energy and low‐energy data, and the sum of the two sets of data is equal to the total absorption of conventional single‐layer detectors.

### 4.2 Virtual monoenergetic image (VMI, MonoE)

**FIGURE 4:**
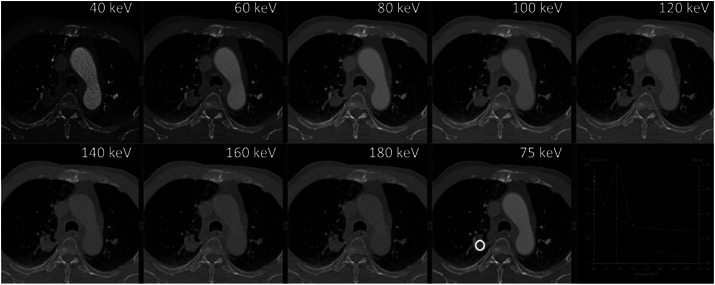
Example of a mono‐energetic reconstruction of a lung cancer patient. Various keV energy levels are reconstructed ranging from 40 keV to 180 keV. The bottom right panel shows the average Hounsfield Unit inside the region of interested together with an estimate of contrast‐to‐noise ratio, showing that 75 keV was optimal for this patient.

Equivalent to the imaging of a single energy ray, the number of images obtained is different based on different acquisition methods, which can generally include a single energy image of 40 to 200 keV. Lower energy levels of VMI can improve the visibility of iodine, potentially resulting in improved identification of lesions containing iodine (Figures 4), thus increasing their visibility and detectability.^4^, ^9^

### 4.3 Color‐coded Zeffective images

**FIGURE 5:**
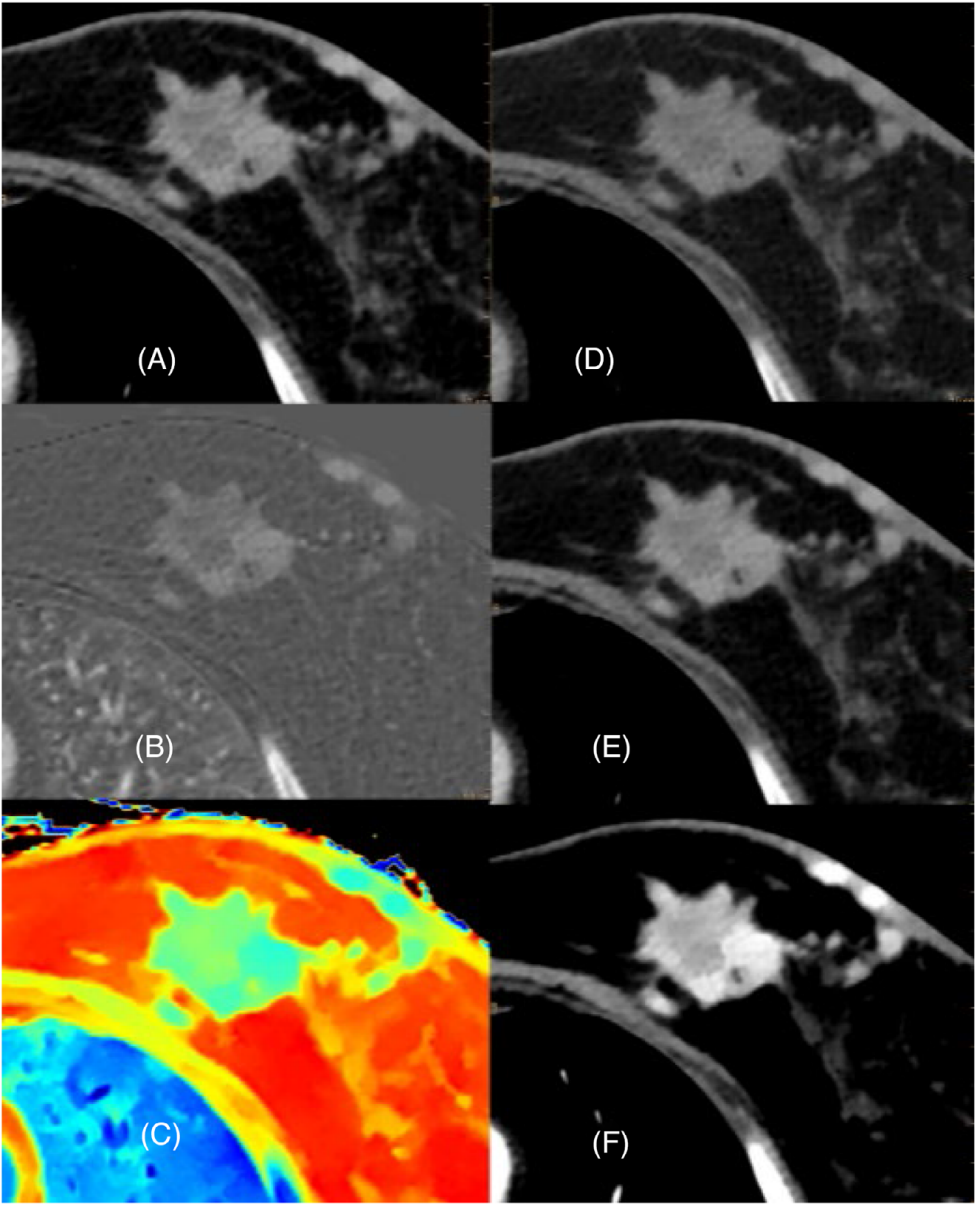
46‐year‐old female patient with recurrent invasive ductal breast cancer. Solid tumor with irregular margins and a mean density of 83.6 HU on the conventional CT‐image (**a**), an iodine‐uptake of 1.39 mg/ml in the iodine‐map (**b**), an elevated Zeffective value of 8.10 on the Zeffective‐map (**c**) and increasing mean densities in the virtual monoenergetic images from 57.9 HU at 100 keV (**d**), over 78.11 HU at 70 keV (**e**) to 161.8 HU at 40 keV (f).

The effective atomic number diagram is also a unique image of dual‐energy CT. The basic principle is to quantify the effective atomic number of each voxel to form a pseudo‐color graph, which can quantitatively determine the effective atomic number (Zeff) and normalized effective atomic number (nZeff, were divided by the effective atomic number of aorta). Color‐coded Zeffective images are able to differentiate between various tissues by using their effective atomic number. This method has been proven to be more effective than comparing Hounsfield Units (HU) on traditional CT scans.^2^, ^4^, ^9^ The application of DECT in breast diseases has attracted increasing attention (Figures 5).^10^

### 4.4 Iodine density map

The commonly used parameters are iodine density map, which can directly show the distribution of iodine and quantitatively determine the iodine concentration (IC) and normalized iodine concentration (NIC, were divided by the iodine concentration of aorta) in the area of interest. The iodine density map is advantageous in not only highlighting lesions that exhibit higher iodine absorption following contrast enhancement, but also in effectively visualizing lesions with lower iodine content.^4^, ^11^

### 4.5 Virtual non‐contrast imaging (VNC)

The iodine‐containing tissue was deionized to make it equal to the CT value without iodine as much as possible to generate an image similar to conventional plain scan, instead of plain scan to reduce the radiation dose received by patients.

### 4.6 The spectral Hounsfield unit curve

Taking the CT value as the ordinate and the single energy level as the abscissa, the material specific curve is obtained, which represents the change characteristics of the CT value of different material components with the energy level. The slope of the spectral Hounsfield unit curve (λ_Hu,_ in Hounsfield unit per kiloelectron‐volt) was calculated as follows:λ_Hu_ = (Hu40keV‐Hu70keV)/30keV.^12^ The composition difference between the focus and normal tissue can be distinguished according to the shape and slope of the curve.

## 5 DECT IN BREAST CANCER DIAGNOSIS

### 5.1 Differential diagnosis of tumor lesions

It is crucial to accurately identify the nature of breast tumors in order to avoid unnecessary biopsies for non‐cancerous tumors and make informed decisions about treatment, which can help reduce costs and complications. By analyzing the shape, enhancement pattern, and blood flow of breast lesions in CT scans with contrast, it is possible to distinguish between benign and malignant tumors.^13^ DECT shows potential in oncological imaging for accurately characterizing tumors.^14^

Several studies have examined the DECT findings of breast lesions and have found promising results in distinguishing between malignant and non‐malignant lesions. Zhang et al^15^ discovered that DECT showed clear benefits in illustrating the invasion of the pectoralis major muscle and the metastasis of axillary lymph nodes. Begüm et al^10^ proposes that the combination of various quantitative parameters such as iodine maps, λ _Hu_, and nZeff in DECT datasets can be used to determine whether breast masses are benign or malignant. They showed that malignant masses have a significantly higher iodine content compared to benign lesions, with a threshold of 0.73 mg/ml and 0.88 mg. In addition, Wang et al^16^ discovered that the quantitative measurements obtained from DECT during both the arterial and venous phases can be utilized to differentiate between benign and malignant breast lesions. NIC, λ _Hu_, nZeff and attenuation of malignant breast lesions are higher than those of benign lesions. In the study of DECT quantitative parameters to distinguish benign and malignant breast tumors, Lan et al^17^ found that except for nZeff, 11 quantitative parameters of malignant tumours were higher than those of benign tumours. In univariate analysis, the venous phase λ_Hu_ is the most effective in differentiating between benign and malignant breast masses. From a clinical point of view, DECT quantitative parameters can be used to distinguish between benign focal findings and malignant focal findings, thus helping to avoid unnecessary follow‐up examinations.

### 5.2 Preoperative diagnosis of metastatic sentinel lymph nodes

Imaging assessments may be used as a complementary noninvasive alternative method for determining the stage of lymph nodes.^18^ The primary objective of preoperative imaging in individuals with breast cancer is to detect the existence of axillary lymph node metastases, which assists the surgeon in determining whether to proceed with axillary lymph node dissection immediately. DECT has a good application prospect in preoperative identification of sentinel lymph node metastasis in breast cancer patients, which can be used as a supplementary non‐invasive method. In a study,^12^ the online arterial phase λ_Hu_ and NIC, the venous phase λ_Hu_, NIC, and nZeff of metastatic sentinel lymph node were higher than those of nonmetastatic sentinel lymph nodes. Other studies^12^, ^19^ have also shown that λ_Hu_ at venous phase obtained through DECT had a high diagnostic performance for the preoperative diagnosis of sentinel lymph node metastases in participants with breast cancer. Zhang et al^15^ also found axillary lymph node metastasis and primary tumor λ _Hu_ were basically the same. In addition, some researchers^20^ also found that there is a parameter similarity in delayed‐phase DECT between primary lesions and lymph nodes in breast cancer, which may be more valuable for predicting lymph node metastasis than simple DECT parameters. DECT shows promise as a quantitative method for assessing lymph node metastases and has the potential to decrease the necessity for sentinel lymph node biopsy.

### 5.3 Pathological classification, molecular subtype and immunohistochemical biomarkers

The National Comprehension Cancer Network Guidelines (version 1.2019) state that surgery of the sentinel lymph nodes is typically not advised for ductal carcinoma in situ (DCIS), as per their recommendations for breast cancer.^21^ Hence, it is essential to distinguish between DCIS and invasive carcinoma in order to make informed clinical decisions. Wang et al^16^ discovered that the levels of NIC were higher in both the arterial and venous phases in invasive non‐special carcinoma compared to DCIS. One possible explanation for this finding could be that invasive non‐special carcinoma has a higher presence of microvasculature and tumor angiogenesis compared to DCIS, leading to increased blood flow within the tumor^22^, leading to a faster peak enhancement time. The iodine map of DECT is the distribution map of the iodine concentration contained in each voxel, which can be used to quantitatively analyze the degree of tissue enhancement. In addition to using black‐and‐white images, iodine fusion color images can also be used to improve the visualization of iodine‐absorbing tissues. The DECT method appears to be a dependable technique with a strong consensus among observers for accurately determining the stage of invasive breast cancers in the local area. Luca et al^23^ found that the iodine concentration and the iodine concentration ratio of DECT can distinguish invasive ductal carcinoma (invasive lobular carcinoma and ductal carcinoma) from other lesions.

The main driving factors of breast cancer are hormone receptor‐related genes, human epidermal growth factor receptor 2 (HER2) gene cluster and proliferation‐related genes. Based on the similarity of gene expression profiles obtained using hierarchical cluster analysis, breast cancer has been subdivided into luminal A, luminal B, HER2‐enriched and basal‐like types. These types have prognostic significance, such as the luminal A subtype has a better overall prognosis and less recurrence than other subtypes.^24^ Therefore, it is essential to determine the molecular subcategories in order to make informed decisions regarding clinical treatment. Wang et al^16^ found that venous phase NIC, as well as arterial and venous phase nZeff can distinguish the luminal A and non‐luminal A subtypes.

Estrogen receptor (ER), progesterone receptor (PR), HER2 and Ki67 are the most useful immunohistochemical biomarkers of invasive breast cancer. The preliminary results of Krug et al^25^ showed that the quantitative parameters of iodine content and nZeff in DECT can be used to evaluate the expression of hormone receptors in invasive breast cancer. Using DECT system, customized breast fixator and biphasic low‐dose data acquisition scheme, Park et al^26^ confirmed the significant correlation between quantitative CT perfusion data and immunohistochemical biomarkers, and significantly increased perfusion in high‐grade, ER‐negative or HER2‐positive cancers. Lin et al ^27^successfully constructed a DECT quantitative parameter combined with a conventional CT feature model,which has a good evaluation performance on the expression level of Ki‐67 in invasive breast cancer. This result was consistent with previous study in rectal tumors^28^ and laryngeal squamous cell carcinoma.^29^

### 5.4 DECT in breast cancer prognosis

DECT‐generated mono‐energetic images with reduced energy levels have the potential to enhance the identification of radiation‐induced alterations in tissue. A preliminary study^30^ by some researchers found that the response of tumors in early‐stage breast cancer patients undergoing radiation therapy can be anticipated by analyzing the alteration of quantitative characteristics, such as the average CT number, on low energy mono‐energetic images obtained from DECT scans performed during the course of radiation treatment. At present, the application of DECT in evaluating the efficacy of radiotherapy and chemotherapy for breast cancer is still insufficient, and more attention can be paid to this direction in the future.

## 6 DECT IN ENHANCING IMAGE QUALITY

### 6.1 Delineation and characterization

Recently, VMIs derived from DECT systems have been found to be clinically useful by improving lesion conspicuity, decreasing artifacts, reducing radiation doses, and material characterization.

Metin et al^31^ confirmed in 29 patients with breast cancer that compared with VM images obtained at 60,80 and 100 keV, VMI obtained at 40 keV had the highest significance of breast cancer, which was consistent with other study.^32^ And some studies^33^, ^34^ suggest that a low keV VMI series reconstructed using a new monoenergetic reconstruction algorithm based on the DECT data allows substantially better visibility of breast cancer than traditional 120 kVp images. Wang et al^16^ also findings seem to indicate that monoenergetic images reconstruction obtained by DECT is valuable for clinical preoperative evaluation of breast cancer.

### 6.2 Artifact reduction

The artifacts produced by lead wire markers in the radiotherapy localization of breast cancer will lead to the deviation between the actual radiation dose and the calculated dose, resulting in an increase in radiation dose in normal tissues. Due to the change in the material of breast metal clips in recent years, artifacts are less than in previous years, but they may still have an impact on treatment plans. It has previously been shown that artefacts have a radiobiological impact (increase of normal tissue complication probability and decrease of tumor control probability) in treatment planning. A study^35^ found that the DECT VM reconstructions can reduce metal artefacts and significantly improved tumour delineation accuracy.

### 6.3 DECT in improving radiation dose calculation

DECT is increased used in tumor radiology, which has aroused the interest of radiation oncology. One of the benefits of spectral CT in radiotherapy is to improve dose calculation.^36^

DECT measurements exhibit a higher degree of quantification compared to measurements obtained through conventional CT techniques^36^, which can provide a wide variety of well‐defined quantitative parameters such as VMI, Zeff as well as electron density (ED) maps. The use of spectral CT data instead of traditional planning CT for dose planning can enhance the precision of various radiation therapy methods and potentially decrease the requirement for phantom calibration to convert HU to ED values.^37^ DECT has the potential to reduce the root mean square errors (RMSE) of proton stopping power ratio (SPR) from 3% to less than 1% uncertainty in proton beam therapy.

## 7 DISCUSSION AND PROSPECT

### 7.1 The advantages of DECT in the diagnosis and treatment of breast cancer

In this review, we briefly summarize the high diagnostic performance of DECT in breast cancer (Table 1) .

**TABLE 1:** Dual‐Energy CT Applications Summary

Application value	Examples of clinical applications
To distinguish benign and malignant breast tumors	Demonstrating the pectoralis major muscle invasion and axillary lymph node metastasis.^15^ The iodine content of malignant masses was significantly higher than that of benign lesions in iodine‐maps.^10^ Quantitative parameters of DECT may allow estimating the benign or malignant nature of breast masses, such as NIC, λ _Hu_, nZeff.^10^, ^15^, ^17^, ^38^ When determining the difference between benign and malignant breast lesions or metastatic lymph nodes, venous phase λ_Hu_ has the highest diagnostic value.^12^, ^17^, ^19^
To provide as a non‐invasive alternate method for staging lymph nodes	To diagnose axillary lymph node metastasis in breast cancer patients.^12^, ^19^ The parameter of primary breast cancer and lymph nodes in DECT images were similar.^15^, ^20^
To assess tumor conspicuity	VMI obtained at 40 keV had the highest significance of breast cancer.^31^, ^32^ The preoperative assessment of breast cancer may benefit from the new monoenergetic reconstruction algorithm of DECT.^16^, ^33^, ^34^
To help breast cancer adjuvant treatment	Potential for distinguishing pathological classification of breast cancer.^16^, ^23^ To discriminate molecular subtype and immunohistochemical biomarkers of breast cancer.^16^, ^25^, ^26^, ^27^
To evaluate the response to treatment of breast cancer	To predict tumor response during radiation therapy of early‐stage breast cancer.^30^

### 7.2 The shortcomings in the existing technology of dual source CT

Compared with ultrasound, mammography and MRI, DECT is not the main method to evaluate breast masses due to the risk of low contrast resolution and exposure to radiation. Compared with other imaging methods, only a small number of literatures have studied the application of DECT in breast cancer. From a scientific point of view, it will be meaningful to compare different imaging techniques, such as diagnostic chest CT, special breast CT and enhanced mammography on the diagnostic effect and radiation dose of breast masses.

A study^39^ showed that DECT has limited potential for differentiating DCIS and Invasive Ductal Carcinoma. DECT does not have the capability to differentiate between DCIS and benign lesions, which is a diagnostic challenge that can be resolved through the use of MRI. Currently, breast MRI is the most precise and reliable method for for locoregional staging of breast cancer.

The results cannot be generalized because only one unique machine was not employed in the present literature. Therefore, more research with various DECT devices and reconstruction techniques is required to thoroughly examine the benefits and drawbacks of each environment.

## 8 CONCLUSION

There are still some deficiencies in the research of the existing literature. Further research is needed to fully incorporate DECT into regular clinical practice as it is still developing. These challenges include as follows:

‐ The premise of precision radiotherapy is the accurate location and staging of tumors. Imaging diagnosis plays a very important role in precision radiotherapy. DECT, a new functional imaging diagnostic technology, has great potential for us to explore in personalized treatment of breast cancer, such as precision radiotherapy.
‐ Predicting response to radiotherapy and chemotherapy. At present, the application of DECT in evaluating the efficacy of radiotherapy and chemotherapy for breast cancer is still insufficient, and more attention can be paid to this direction in the future.
‐ Increasing the positive predictive value and consistency of molecular subtypes. The quantitative parameters of DECT can predict molecular subtypes of breast cancer, but lack of good interobserver agreement.
‐ Combining DECT imaging with radiomics to improve the diagnosis, prognosis, prediction and monitoring of breast cancer. In the current era of personalized medicine, the field of radiomics holds promise for enhancing various aspects of breast cancer management, including diagnosis, prognosis, prediction, monitoring, image‐guided interventions, and evaluation of treatment response^40^.
‐ Establishing a model combining the Iodine density‐maps, Color‐coded Zeffective images and ER status was established to predict tumor regression patterns after neoadjuvant therapy at a very early stage, which will provide individualized diagnosis and treatment of breast cancer after neoadjuvant therapy. It is very important to judge whether the tumor is sensitive to neoadjuvant therapy at early stage, but conventional and functional breast imaging techniques are incapable of accurate prediction of residual disease.
‐ Studying the feasibility of VNC replacing true non‐contrast imaging in breast cancer treatment planning. Radiotherapy plans for breast cancer generally need to be made on non‐contrast images. VNC may reduce the radiation dose and economic cost by reducing one scan of the patient.
‐ Using DECT and reconstruction algorithms to reduce the metal clip artifacts of breast radiotherapy, and improve the accuracy of tumour delineation and radiation dose.

The quantitative parameters based on the spectral imaging data of dual‐energy CT have been proved to be applicable to the diagnosis of several types of tumors. Dual‐energy CT can be used as a supplementary noninvasive method for breast cancer, and it has great value in the diagnosis, treatment strategy and prognosis prediction of breast cancer in the future.

## CONFLICT OF INTEREST STATEMENT

The authors declare no conflicts of interest.

## ETHICS STATEMENT

None.
